# Inflammatory disease of the costotransverse joints: US evaluation in 15 symptomatic patients

**DOI:** 10.1007/s40477-021-00589-5

**Published:** 2021-06-12

**Authors:** A. Del Chiaro, B. Ciampi, F. Franzoni, M. Miccoli, S. Galletti, S. M. Stella

**Affiliations:** 1 Advanced Musculoskeletal Ultrasound, SIUMB School of Pisa, Pisa, Italy; 2 Orthopedic and Trauma Operating Unit, University of Pisa, Pisa, Italy; 3 Advanced Musculoskeletal Ultrasound SIUMB School of Bologna, Maggiore Hospital, Bologna, Italy; 4 School of Specialization in Sports Medicine, Department of Clinical and Experimental Medicine, University of Pisa, Santa Chiara Hospital of Pisa, Pisa, Italy; 5 Department of Clinical and Experimental Medicine, University of Pisa, Pisa, Italy

**Keywords:** Costotransverse joint, Costotransverse joint anatomy, Costotransverse joint biomechanics, Musculoskeletal ultrasound, Thoracic back pain

## Abstract

The costotransverse joints (CTJs) are small arthrodial joints which articulate with the costal tuberosity on the transverse process of the thoracic vertebrae. CTJs are composed of oval-shaped facets with a major axis, vertical at the upper vertebrae and almost horizontal at the lower vertebrae. This position explains the different movements of the ribs: the cranial ribs move on the sagittal plane and the caudal ribs on the transverse plane. Movements in directions other than these usual CTJ spatial planes can cause inflammation resulting in a stinging pain in the space between the scapula and thoracic spine. We studied 15 subjects with paravertebral pain compatible with CTJ pathology. Mean age was 29 years, 11 females/4 males. In 12 patients, the non-dominant limb was affected. US imaging was carried out using linear 12 MHz and 9 MHz probes. Scanning was performed following the long axis of the rib (transverse plane) and the short axis (sagittal plane). Sagittal scanning is the method of choice for detection of possible joint effusion and comparison with undamaged joints above and below. US identified joint effusion correlating with the site of pain in all patients. Thickening of the posterior costotransverse capsular ligament was detected in six patients mainly affecting the first thoracic vertebrae. Power Doppler showed intraarticular hypervascularization in four patients. US imaging should be performed as a first-line examination in the evaluation of patients with stinging pain in the paravertebral region. US evidence of effusion within the joints is a sure sign of involvement of these structures.

## Introduction

The costotransverse joints (CTJs) are anatomical structures which, together with the costovertebral joints, form the connection point between the rib cage and the thoracic vertebrae [1–3]. The CTJs and costovertebral joints also contribute to the stability of the thoracic spine [1–4].

In the group of thoracic spine pathologies, CTJs are no doubt less studied than the joints located between the intervertebral facets and those between the vertebral bodies and fibrocartilaginous discs [5–8]. CTJs can cause a particular form of “thoracic back pain” which may be difficult to classify in clinical practice [9–12].

The purpose of this study of 15 symptomatic patients was to provide directions for accurate ultrasound (US) imaging of these small joints through the study of their anatomy and biomechanics. This knowledge is essential for the exact understanding of the pathophysiological mechanisms underlying inflammation of the arthrodial joints. Moreover, considering the deep location of the CTJs an accurate US study facilitates possible US-guided infiltration therapies.

## Materials and methods

### Normal anatomy and biomechanics

**Fig.1:**
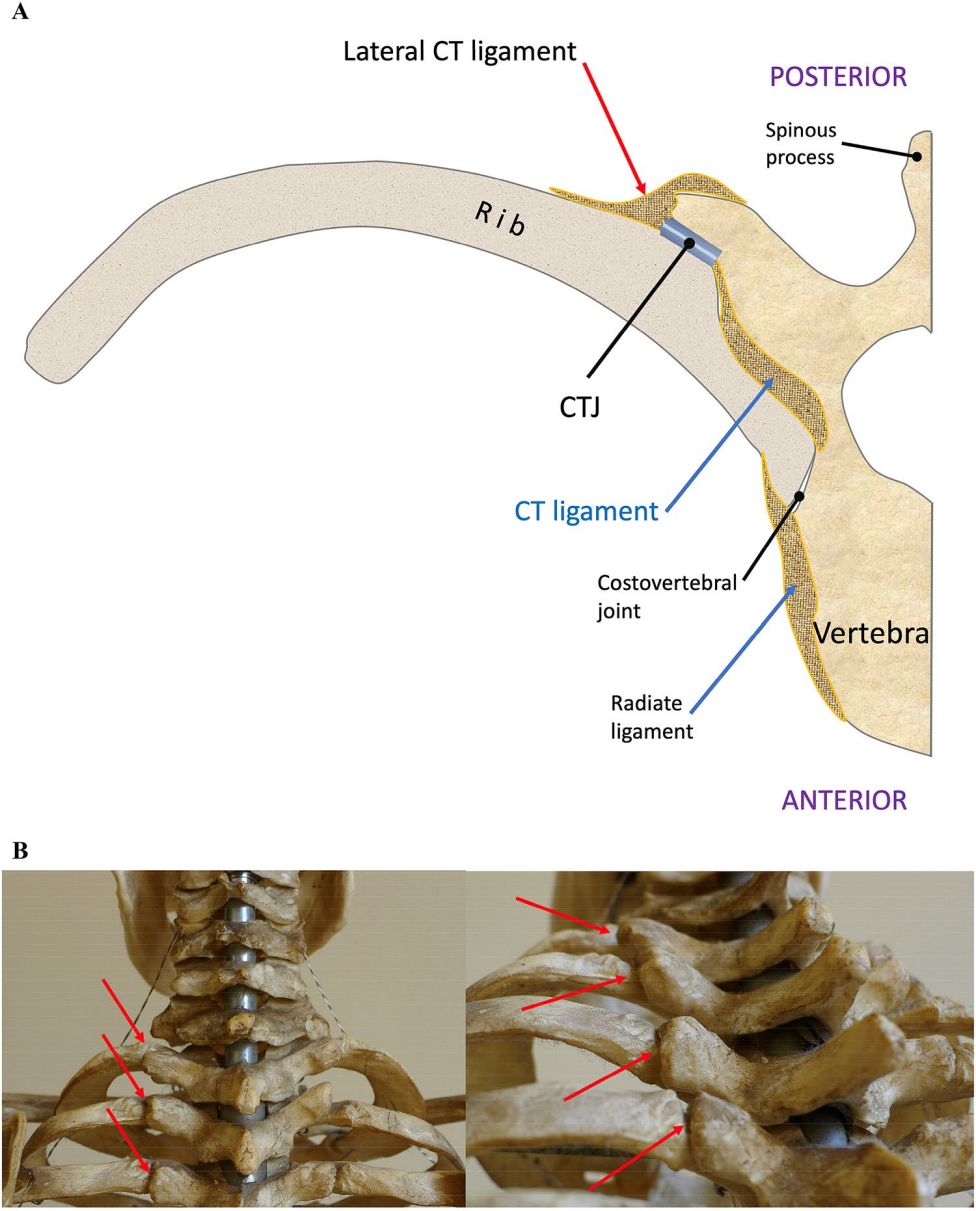
**a** Schematic drawing illustrating the right half of a thoracic vertebra (transverse section) resected at the level of the body, articulated with the rib in front of the costal angle: the costotransverse joint (CTJ), the costotransverse ligament (CT), the lateral costotransverse ligament and the radiate ligament are shown. **b** Dorsal view of the upper rib cage of a skeleton with the first CTJs (red arrows)

The ribs articulate with the vertebral column through two small gliding joints: the CTJs and the costovertebral joints. The CTJs, which are simple arthrodial joints, are located between the costal tuberosity and the transverse process of the thoracic vertebrae (Fig. 1) but not at the 11th and 12th ribs (floating ribs).

**Fig. 2:**
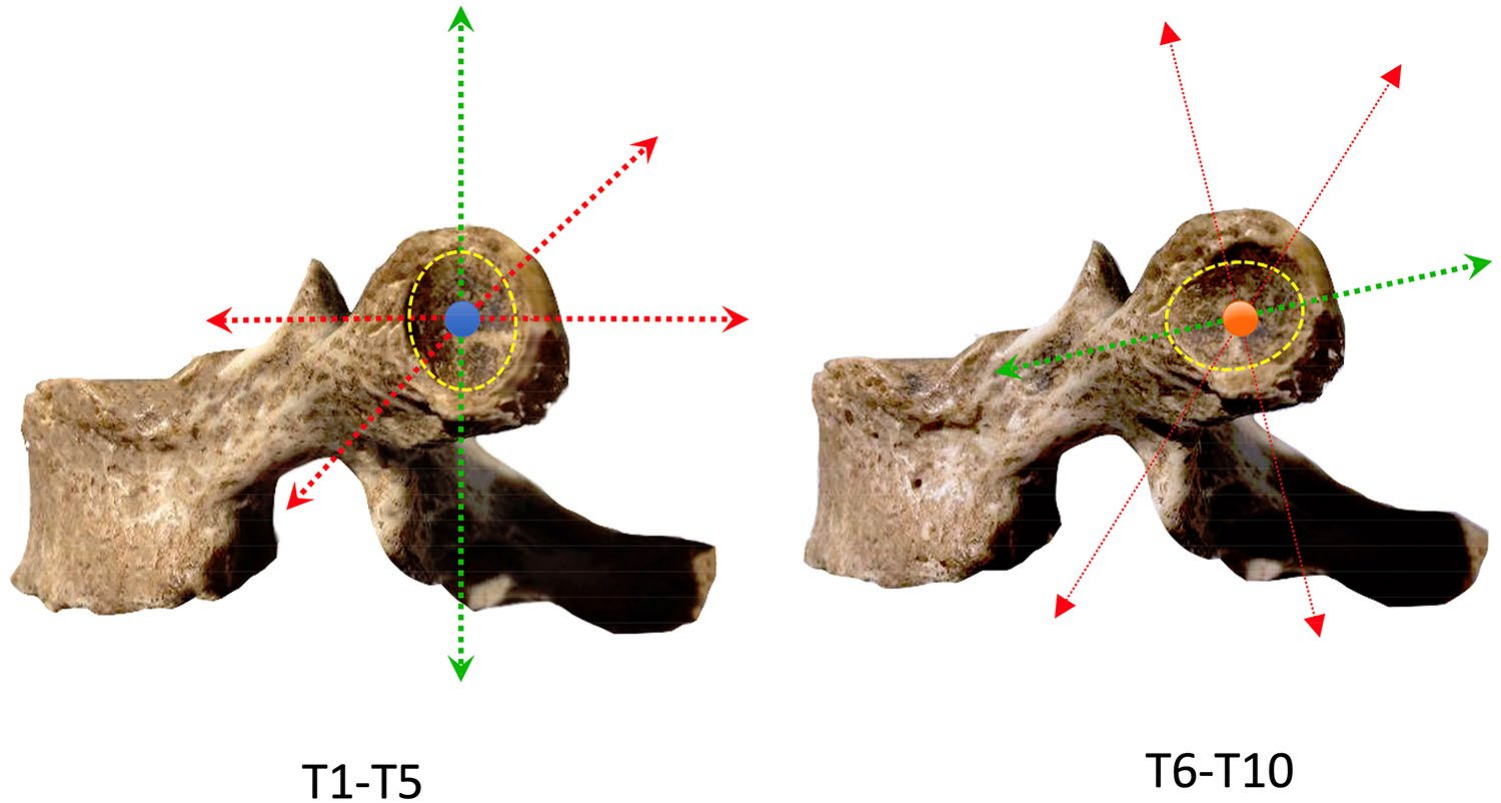
The articular surface of CTJ is convex on the rib side and concave on the vertebral side. The direction of the major axis of the arthrodial joints is different when proceeding from the more cranial joints compared to the caudal ones. The vertebrae from T1 to T5 have a joint that is spatially oriented with a vertical major axis, while the orientation of the joint facets of vertebrae T6–T10 is almost horizontal, slightly inclined anteriorly and downwards. The figure shows the force vectors for T1–T5 (red dashed arrows) contrary (orthogonal or oblique) to the vertical orientation (green arrow) of these arthrodias: stress is put on the joint when muscle forces act on the transverse or oblique plane (red dashed arrows), but this may also occur with overuse on its "natural" sagittal plane (green dashed arrows). Same concept can be applied to the distal arthrodias (T6–T10); however, these joints are rarely affected by pathology

The articular surface is convex on the rib side and concave on the vertebral side. The direction of the major axis of the arthrodial joints is different when proceeding from the more cranial joints compared to the caudal ones: the vertebrae from T1 to T5 have a joint that is spatially oriented with a vertical major axis, while the orientation of the joint facets of vertebrae T6–T10 is almost horizontal (slightly inclined anteriorly and downwards) [13] (Fig. 2).

This spatial arrangement of the upper gliding joints is different from that of the lower ones, thus reflecting the different movement of the first five ribs compared to the last five: the upper ones move on the sagittal plane and the lower ones on the transverse plane.

The CTJs are completed by a joint capsule which in turn has several reinforcing ligaments (Fig. 1) [14]: (a) the intrinsic interosseous ligament or transverse ligament of the cervical vertebra running from the transverse process to the posterior wall of the rib neck; (b) the superior costotransverse ligament running from the lower edge of the transverse process to the upper edge of the underlying rib neck; (c) the lateral costotransverse ligament or posterior costotransverse ligament running from the apex of the vertebral transverse process to the external portion of the costal tuberosity.

The costovertebral joint is a small arthrodial joint situated between the head of the rib and two adjacent vertebral bodies; it has a joint capsule and a triangular-shaped reinforcing ligament (radiate ligament).

**Fig.3:**
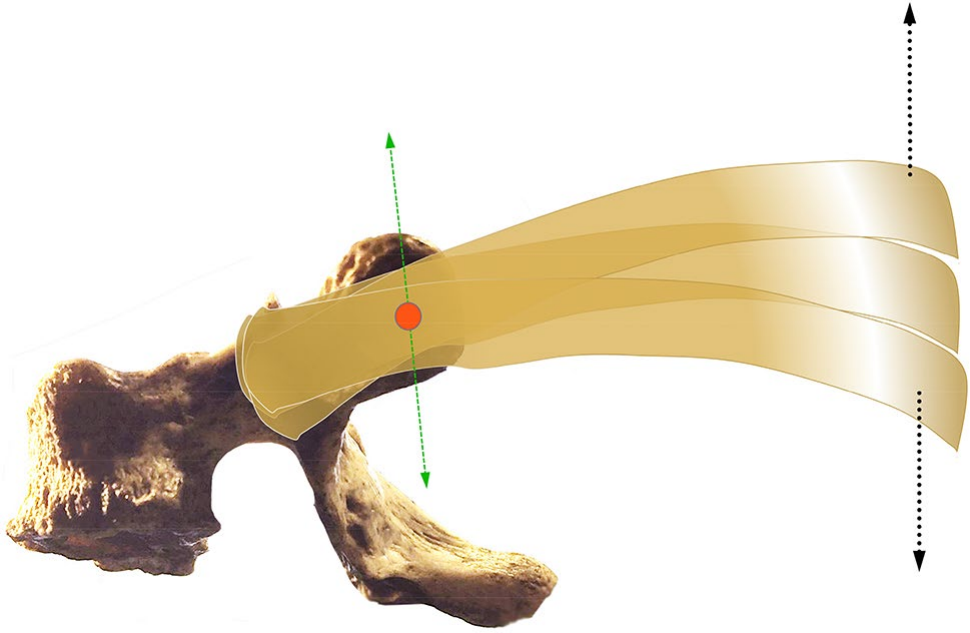
The costovertebral joints and the CTJs cannot operate independently as they are closely connected by a mechanical constraint similar to a first-class lever system. The fulcrum (red dot) is the CTJ and the two symmetrical levers are the head of the rib and the posterior vertebral body. The schematic drawing shows the movements of the first five ribs occurring on a sagittal plane (black arrows) during breathing. Green arrows indicate the vectors of ‘physiological’ motion of the CTJs

The costovertebral joints and the CTJs cannot operate independently as they are closely connected by a mechanical constraint similar to a first-class lever system (where the fulcrum is the CTJ and the two symmetrical levers are the head of the rib and the posterior vertebral body) (Fig. 3).

The forces acting on the CTJs can be divided into extrinsic and intrinsic forces. The extrinsic force is mainly compression on the anterior rib cage (e.g., compressive trauma caused by a car seat belt in case of a collision) [15].

The intrinsic forces are the tractions exerted by the major muscle groups that are inserted on the ribs or on the thoracic vertebrae. The muscle bellies inserted on the ribs, which determine the movements of the CTJs, are essentially those of the respiratory muscles (internal and external intercostal, elevator of the ribs, scalene and sternocleidomastoid, serratus anterior and posterior).

Activation of these muscles causes motions on the sagittal plane of the ribs and this may have a direct impact on the CTJ. The various other muscles insert on the thoracic vertebrae at the spinous processes or at the transverse processes.

**Fig.4:**
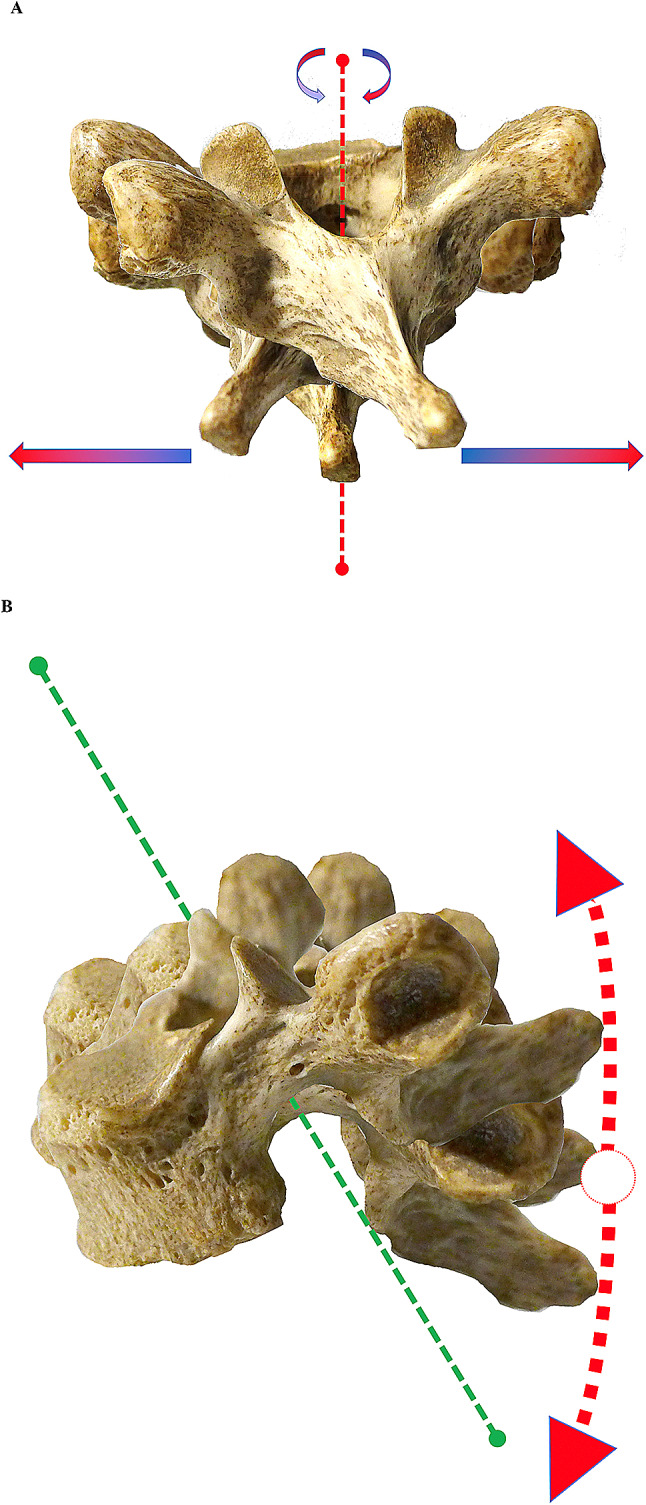
**a** Biomechanics of CTJs: movements that the thoracic vertebrae undergo in the *transverse plane* due to the action of various muscles that are inserted on the spinous processes and on the transverse processes of the vertebrae. **b** Movements in the sagittal plane due to the action of various muscles that are inserted on the spinous processes and on the transverse processes of the vertebrae. However, the movements in this plane are limited by the presence of the nearby vertebrae and by the intervertebral discs

The main muscles inserted on the thoracic vertebrae (on the spinous processes) include the trapezius and rhomboid muscles (major and minor). They permit motion on the axial plane that is opposite to the predominant plane of the more cranial CTJs. The muscles of the intermediate plane, the nuchal muscles (the semispinalis muscles of the head, neck and back) that extend and rotate the vertebral column are inserted on the transverse processes, stressing the joint mainly on the sagittal plane. Considering the main activity of these muscles, it appears evident that repeated movements of the shoulder (especially flexion, external rotation and abduction), of the head (especially flexion–extension and rotation), of the scapula (abduction and adduction) and of the thoracic spine (torsion) can cause inflammation due to overuse of the CTJ [15](Fig. 4).

The same type of stress can also be caused by activities triggering deep and fast breathing (e.g., competitive sports but also restrictive and/or obstructive pulmonary diseases) [15].

### US scanning technique and patients and methods

The examination is carried out using high-density multifrequency linear probes. It may be difficult in obese patients or in patients with particularly developed muscle masses such as body-builders. In these patients, the joint lies deeper than in lean subjects [16] and probes with a lower frequency, 7.5–10 MHz or linear 9 MHz probes, are, therefore, required. The focus must be placed at the level of the joint plane with compound and tissue harmonic imaging (THI) activated to identify possible fluid collections in the small joint capsules. US imaging must always be performed on two planes: (1) a plane on the major axis of the rib (long axis) that corresponds to a plane section along the transverse axis, and (2) a plane on the short axis of the rib (short axis) that corresponds to a sagittal plane.

**Fig.5:**
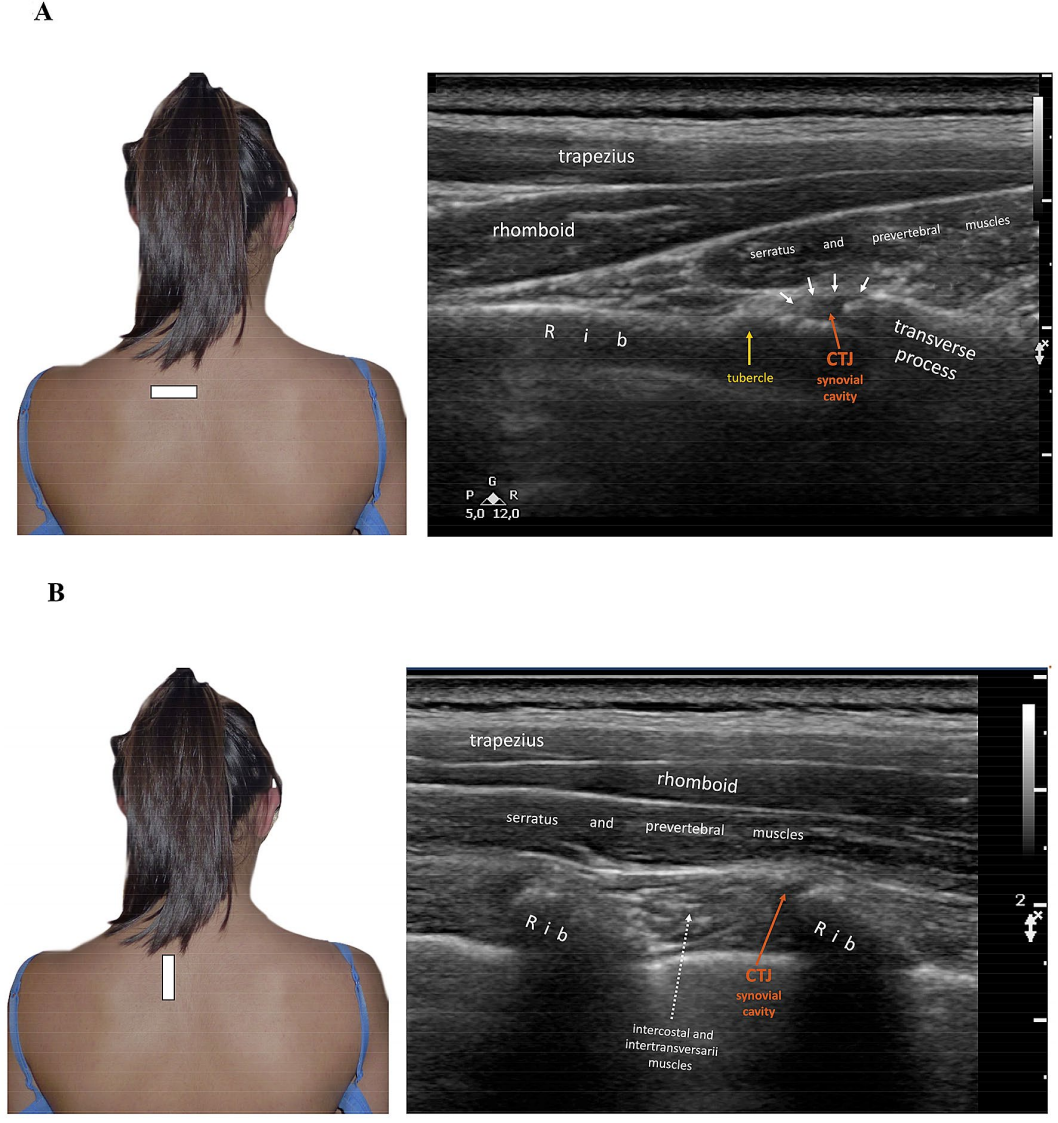
**a** US image of the CTJs with the probe on the major axis of the rib (long axis). White arrows indicate the lateral or posterior costotransverse ligament. **b** Probe on the short axis of the rib: the short axis is useful for identifying joint effusion and makes it possible to compare two/three adjacent joints simultaneously to detect pathological differences

**Fig.6:**
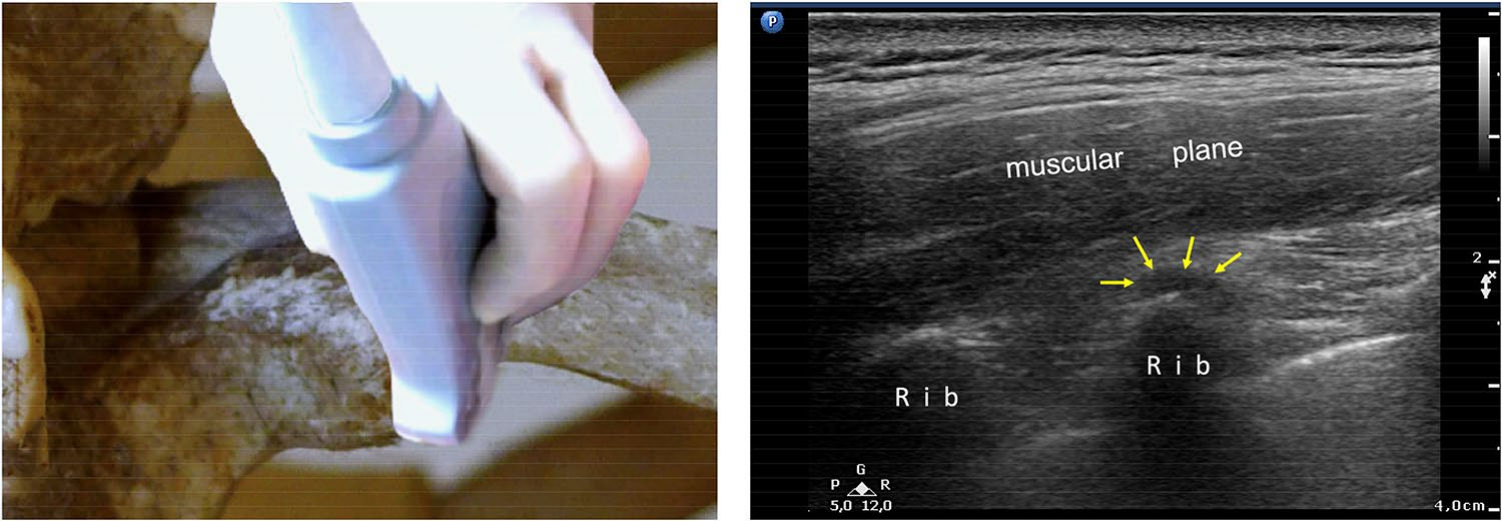
The short axis is the most useful for identifying joint effusion: yellow arrows show joint anechoic effusion in the synovial cavity

The short axis is very useful for identifying possible joint effusion and joint inflammation. This scan makes it possible to compare two or three adjacent joints simultaneously (depending on the length of the probe) to detect pathological differences (Figs. 5 and 6).

Not all the anatomical structures of the CTJs are visible at US imaging. However, the joint capsule is easily identified (it usually appears relaxed and without effusion) and so is the lateral or posterior costotransverse ligament (which may be thickened if affected by inflammation) (Fig. 5).

To define the affected CTJ, it is necessary to identify the spinous process of the vertebrae which is located on the same plane section. Due to the particular inclination of the spinous processes of the thoracic vertebrae, the level of the affected CTJ can be correlated with that of the spinous process of the overlying vertebra.

If, for example, the affected joint is the one located at the level of the spinous process of T3, the pathological CTJ will be the one of the fourth ribs and the vertebra will be T4. In clinical practice, the spinous processes are easily identified starting to count them from the 7th cervical vertebra which is denominated "prominent" because of the particular length of its spinous process, which is easily identified also in obese patients.

**Fig.7:**
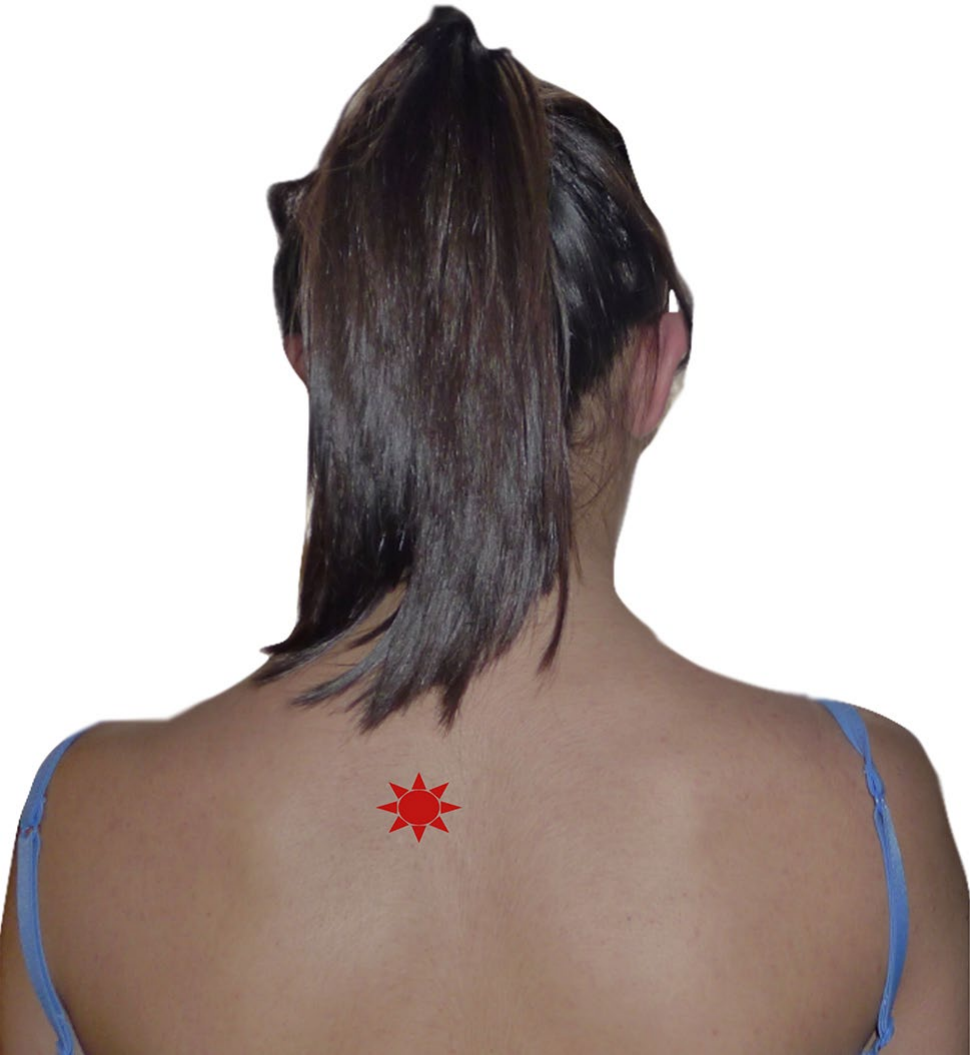
Area of the proximal paravertebral region (most often of the non-dominant side) where stinging and burning pain is reported

A total of 15 patients were referred to our section due to stinging and burning pain (Fig. 7) limited to the paravertebral region compatible with costotransverse joint pathology; all underwent US imaging in a standing position. US imaging was carried out by two operators using Philips HD 15 and a multifrequency linear transducer (12 MHz) or a GE EQ9 and a matrix linear probe (6–15 MHz) or a 9-MHz linear multifrequency probe. The focus depth was set to the joint plane and compound and THI were activated. The CTJs were evidenced in all patients. In three patients (3/15) the 12 MHz probe did not provide adequate visualization of the joints, so a 9 MHz probe was used. In all patients, the joint was studied on both sagittal and transverse scans. Demographic and clinical data as well as written informed consent were collected from all patients.

### Statistical analysis

Descriptive statistics are reported as means, absolute frequencies and percentages. Cohen’s kappa coefficient was calculated to evaluate the agreement between the operators. Haldane–Anscombe correction, commonly used to avoid a division by zero in the calculation of odds ratio, was applied by adding 0.5 to each cell of the frequency table. The Haldane–Anscombe method was used in an unreleased way in the Cohen's kappa calculation. Cicchetti’s guideline was adopted to interpret the kappa coefficient: values between 0.40 and 0.59 are associated with a fair agreement.

Statistical software R 4.0.3 was used to perform the analysis.

## Results

The demographic data of the studied patient population are summarized in Table 1. Mean age of the enrolled patients was 29 years. Most of them (73.3%) were females. The most frequently affected limb was the non-dominant one (80% of cases) (Fig. 7). US findings are summarized in Table 2.

**Table 1:** Graphic summary of the population demographic data

Characteristics	Frequency
Mean age (years)	28.8 ± 3.7 (18–32)
Gender	11 F: 4 M
Dominant limb: non-dominant	3: 12

**Table 2:** Graphic summary of ultrasound findings in studied population

Characteristics	Patients (%)
Joint effusion	15 (100)
Affecting D1–D4	11 (73)
Affecting D5–D10	4 (27)
Correlation with pain and joint effusion	15 (100)
Posterior costotransverse ligament thickening	6 (40)
Power Doppler signal	4 (27)

**Fig.8:**
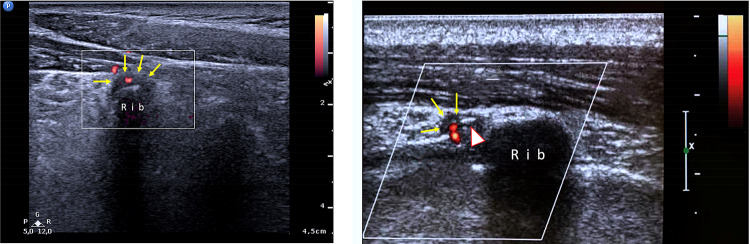
Presence of Doppler US signal in the synovial tissue (yellow arrows) in less than half of the area of the synovium (27% of cases): these findings were detected mainly in chronic inflammation; white and red arrowhead show a little calcification within the CTJ

In all patients presenting with hypo-anechoic effusion within the costotransverse joints, the finding was correlated with the sites where the patients reported pain. In 73% of cases, the four most cranial joints were involved (Fig. 7). Less frequent findings were thickening of the posterior costotransverse ligament (40% of cases) and the presence of Doppler US signal in synovial tissue in less than half of the area of the synovium (27% of cases) (Fig. 8); these findings were detected mainly in chronic inflammation (duration of symptoms > 6–8 weeks).

The Cohen's kappa value, calculated to evaluate the agreement between the operators, is 0.47, assuring a fair inter-rater reliability.

## Discussion

Pain syndrome caused by inflammation of the costotransverse joints is one of the possible differential diagnoses in patients with "thoracic back pain" in addition to the pain syndrome deriving from the joints located between the intervertebral facets and intervertebral discs [9–12].

The clinical symptoms caused by inflammation of the CTJs are quite characteristic: a stabbing pain sometimes associated with a burning soreness like most cases of somatic pain located in the paravertebral region (between the scapula and the thoracic spine) [5, 11] (Fig. 7).

In our experience, this painful CTJ syndrome develops more frequently in young subjects (probably due to the greater muscle mass and, therefore, the greater forces acting on the CTJs), more often in females and in the non-dominant side (probably because these joints are usually subject to less movement showing that also a limited stress intensity may be the origin of this pathology) and it affects mainly the first four/five thoracic vertebrae which have CTJs with a longer vertical axis of the articulation moving mainly on the sagittal plane [3].

Not infrequently, anterior radiation of pain may reach the lateral surface of the rib cage. The pathogenesis of this phenomenon is not yet fully understood. Some authors hypothesize that it is linked to the combination of CTJ and costovertebral inflammation [9], but in our opinion, the phenomenon can also be attributed to a possible irritation of the intercostal nerves [3].

Irritation of the posterior branches of the thoracic spinal nerves can also account for a burning nature of the pain. Pain deriving from the CTJs presents the peculiar characteristics of a sharp pain and paravertebral localization between the medial edge of the scapula and the spinous processes of the thoracic vertebrae [5–11].

The purpose of this study was to provide anatomical and biomechanical clues to help identify the clinical characteristics of the painful CTJ syndrome to plan adequate therapy. The most recent studies reported in the literature addressing inflammation of CTJs state that the pathophysiology of this disorder is not yet completely clear [9, 17].

A CTJ functions as a sort of "hinge" between the rib cage and the dorsal spine and it is, therefore, subject to numerous forces that can lead to abnormal movements.

The forces acting on these arthrodial joints can be extrinsic, e.g., anterior chest compression or traumatic rib injury, and intrinsic, e.g., muscle insertions stressing the thoracic vertebra on the spinous process or directly due to insertions on the CTJs acting with force vectors (orthogonal or oblique) opposed to the vertical orientation of the proximal gliding joints, thus stressing the joint on the transverse or oblique plane (Figs. 2, 3 and 4).

These forces act on the vertebral body generating motions both on the axial plane (in an opposite direction with respect to the spatial orientation of the arthrodial joint) and on the sagittal plane by acting on the spinous processes, although the sagittal plane motion is limited by the nearby vertebrae and the intervertebral discs. Finally, also heavy breathing can act on the joint causing a rotational motion of the arthrodial joint on the sagittal plane (however, this is a 'natural' motion which follows the vertical orientation of the arthrodial joint).

If protracted over time, the described motions may develop overuse and subsequent inflammation in these joints. On the basis of these premises, both flexion, extension and rotation movements of the neck, and particularly scapular abduction and adduction, shoulder flexion, external rotation and abduction, torsion movements of the thoracic spine, neck movements such as rotation of the head and heavy breathing (especially during competitive physical activity, but also in patients with pulmonary pathologies) can have an impact on the vertebral bodies of the thoracic vertebrae causing a certain degree of stress (rotation) on the various costotransverse axial planes.

The greater the muscle mass, the greater is the stress and muscle trophism involved in these movements and this may account for the greater incidence of pain syndrome in young subjects.

The prevalence of pain syndrome involving the non-dominant side is likely to be linked to a reduced movement of the limb (a joint that moves little reacts to maximum stress in a very different way compared to a joint accustomed to movement) and perhaps also to reduced coordination of the various muscle groups of the non-dominant limb of this side of the human body, compared to the dominant side.

This diagnostic study involving 15 patients referred to us with symptoms typical of inflammation affecting the CTJ showed that this painful syndrome occurs more frequently in women. It can, therefore, be hypothesized that the prevalent involvement of the first four CTJs is linked to their morphology.

Another important part of this study is that the typical US characteristics indicating CTJ pathologies have been identified. The limitation of the study is linked to the small patient population; however, in 100% of cases CTJ inflammation developed fluid collection within the small joint capsules.

Another interesting fact is the exact correlation between the site of pain pointed out by the patient and the presence of CTJ fluid collection (100% of cases).

Posterior costotransverse ligament thickening and joint capsule hypervascularity were less frequent (about 1/3 of cases). These findings are generally linked to chronic inflammation or diagnosed in patients with persistence of symptoms for > 5–6 weeks and only partial recovery despite oral or intramuscular anti-inflammatory therapy.

## Conclusions

US may become the instrumental examination of choice in the evaluation of patients who have stinging and burning pain in the paravertebral region between the medial border of the scapula and the spinous processes of the thoracic vertebrae. The presence of a CTJ capsule fluid collection is the main US sign of pathological involvement of the joint.

Posterior costotransverse ligament thickening and joint capsule low-level power Doppler signal are characteristic findings only in cases of chronic inflammation. US guidance may be also essential in anti-inflammatory small joint infiltration [9].

## Data Availability

All data are available for any further revision.
